# Effectiveness of power training compared to strength training in older adults: a systematic review and meta-analysis

**DOI:** 10.1186/s11556-022-00297-x

**Published:** 2022-08-11

**Authors:** Mohamed el Hadouchi, Henri Kiers, Ralph de Vries, Cindy Veenhof, Jaap van Dieën

**Affiliations:** 1 Institute for Human Movement Studies, University of Applied Sciences Utrecht, Heidelberglaan 7, 3584 CS Utrecht, The Netherlands; 2 Department of Human Movement Sciences, Vrije Universiteit Amsterdam, Van der Boechorststraat 7, 1081 BT Amsterdam, the Netherlands; 3 Research Group Innovation of Movement Care, University of Applied Sciences Utrecht, Heidelberglaan 7, 3584 CS Utrecht, the Netherlands; 4 Department of Rehabilitation, Physical Therapy Science and Sport, Brain Center, University Medical Center Utrecht, Heidelberglaan 100, 3584 CX Utrecht, the Netherlands

**Keywords:** Muscle power, Muscle strength, Activity tests

## Abstract

**Background:**

Research suggests that muscle power is a more critical determinant of physical functioning in older adults than muscle strength. The objective of this study was to systematically review the literature on the effect of power training compared to strength training in older adults on tests for muscle power, two groups of activity-based tests under controlled conditions: generic tests and tests with an emphasis on movement speed, and finally, physical activity level in daily life.

**Methods:**

A systematic search for randomized controlled trials comparing effects of power training to strength training in older adults was performed in PubMed, Embase, Ebsco/CINAHL, Ebsco/SPORTDiscus, Wiley/Cochrane Library and Scopus. Risk of bias was assessed using the Cochrane Collaboration Tool, and quality of evidence was evaluated using GRADEpro Guideline Development Tool. Standardized mean differenences (SMD) and 95% confidence intervals (CI) were calculated for outcomes separately using a random effects model.

**Results:**

Fifteen trials and 583 participants were included in the meta-analysis. Results indicated a statistically significant benefit of power training on all reported outcomes (muscle power SMD: 0.99, 95% CI: 0.54 to 1.44, *p* < 0.001; generic activity-based tests SMD: 0.37, 95% CI 0.06 to 0.68; *p* = 0.02, activity-based tests emphasizing movement speed SMD: 0.43, 95% CI 0.23 to 0.62, *p* < 0.001). None of the included studies used physical activity level in daily life as outcome.

**Conclusions:**

Power training offers more potential for improving muscle power and performance on activity tests in older adults compared to strength training. Future research should assess exercise parameters for power training in older adults. In addition, the validity and reliability of the tests used must be evaluated to establish a standardized test protocol. This protocol should also include measurements of physical activity in daily life.

**Supplementary Information:**

The online version contains supplementary material available at 10.1186/s11556-022-00297-x.

## Introduction

The aging process is characterized by the degeneration of various physiological systems, including the neuromuscular system, which may lead to a loss of muscle strength (the ability to produce large muscle force) and muscle power (the ability to produce a large muscle force at high contraction velocity) [1–3]. This decline may cause daily tasks, such as getting up from a chair or climbing stairs, more difficult to perform, often resulting in a loss of independence for older adults. Intervention studies have indicated that both strength and power training can improve functional capacity in older adults, consequently improving their ability to maintain independence [4–9].

Several studies have revealed that the annual decline in muscle power is larger than the annual decline in muscle strength in older adults [10–16]. The actual percentages of annual decline in muscle power and muscle strength varied between studies and were largely dependent on age and sex. However, the conclusion that muscle power declined more rapidly than muscle strength was consistent. Furthermore, in daily activities, such as getting up from a chair, the ability to move with a sufficiently large speed (emphasizing muscle power) is more often the limitating factor than the ability to produce a sufficiently large moment [10, 17]. These findings signify the potential importance of emphasizing muscle power in the training and rehabilitation of older adults instead of focusing on strength [6].

Several studies have shown that training specifically aimed at increasing muscle power can improve the ability to generate high power output even in older adults [7, 17, 18]. Power training consequently also improved physical functioning in daily life. The effects appeared even larger than after strength training and endurance training [3, 4, 7, 8, 17–20].

Two reviews have systematically evaluated the effect of power training in older adults [8, 20], but methodological limitations, namely high hetereogeneity between studies, limited search strategy, variation in outcomes measures, variation in the exercise methods, and non-specific definitions of power training influenced the comparison of power training against strength training.

The aim of this study was to systematically review the literature on the effect of power training compared to strength training in older adults with muscle power, activity-based tests, and physical activity level in daily life as outcomes.

## Methods

### Protocol and registration

The present study was developed in according to Preferred Reporting Items for Systematic Reviews and Meta-Analyses (PRISMA) guidelines [21] and was registered in the International Prospective Register of Systematic Reviews (PROSPERO 2021: CRD42020167877).

### Data sources and search strategy

This review included randomized controlled trials (RCT) that compared a power training intervention with a strength training intervention in older adults. A comprehensive search was performed in collaboration with a medical librarian using the bibliographic databases PubMed, Embase, Ebsco/CINAHL, Ebsco/SPORTDiscus, Wiley/Cochrane Library and Scopus up until 18 September 2020. The search strategy combined thesaurus terms and free texts words. The PubMed search string (Additional file 1) was constructed first and used as a template for the other databases. All references from the selected articles were checked to identify additional relevant articles that were missed in the systematic search. To minimize publication bias, an additional search for grey literature was performed in the open access thesis and dissertation database and the World Health Organization (WHO) international clinical trials registry platform using the search terms “power training”, “physical performance” and “elderly”.

### Study selection

The study selection was performed independently by two co-authors (HK and MeH) in two stages, and regular meetings were organised to form consensus in the selection and scoring of studies within each stage. In the first stage, all abstracts from the systematic search were preliminarily screened on eligibility criteria using the online application ‘Rayyan’ [22]. In the second stage, full text articles were read to ensure that the selected studies met eligibility criteria.

RCTs comparing a power training group with a strength training group were included if they met the following eligibility criteria: (a) the study population consisted of older adults (mean age > 65 years) recruited from a healthy population, regardless of their level of physical functioning. Healthy was defined according to the WHO definition for health, in which individuals can be considered healthy despite the presence of (chronic) disease [23]; (b) the intervention was power training. This was assessed in two ways: (1) the authors defined their intervention as power training; or (2) the intervention met the definition of muscle power training proposed by Haff and colleges [24]: “an intervention primarily aimed at muscle power, movement speed or rate of force development”; (c) the study included outcome measures for muscle power, activity based tests, or a measure for physical functioning in daily life. These measurements had to be performed in a laboratory or clinical setting. We divided these tests in two categories, ‘generic tests’ and test with an emphasis on the speed of execution (‘speed tests’); (d) the strength training control group was age-matched and received at least partially supervised strength training; and (e) studies were published in English, Dutch, or German language. Studies were excluded if the study population consisted solely of participants with specific musculoskeletal, neurological or psychological diseases on the basis of non-generalizability. Studies were also excluded if the interventions were home-based or solely internet-based interventions in view of concerns regarding adherence.

### Data extraction

First, relevant outcome data and participant and intervention characteristics were extracted. Secondly, if required, standard errors were converted to standard deviation for activity tests to allow between-study comparisons. From the selected studies that included a non-training control group in addition to power training and strength training groups, data were extracted for a separate meta-analysis of power training versus a non-training control group to test the assumption that power training is superior to non-training.

### Risk of Bias

The methodological quality of the selected studies was assessed using the Cochrane Collaboration Risk of Bias tool [25]. The category for blinding of outcome assessment was scored as high risk of bias by default due to the difficulty in maintaining true blinding during post-intervention measurements. Studies were upgraded to unclear or low risk of bias if attempts were made to blind the outcome assessment for patients or assessors (e.g. blinding of patients to former assessment). Blinding of participants and personnel was not included in the risk of bias assessment as the nature of the studies does not allow for true blinding of the participants for the intervention that is received. All other types of bias were assessed according to the guidelines in the Cochrane handbook [25]. Studies were considered high risk of bias when three or more items were scored unclear or high, or when two items were scored high.

### Statistical analysis

A meta-analysis was performed comparing the difference in intervention effects for tests measuring muscle power, performance on the two types of activity based tests, and level of physical activity using RevMan 5.3 software (2014). The secondary meta-analysis comparing power training with a non-training control group was performed using the same procedure and software. Meta-analysis results are presented separately for each outcome, in forest plots using standardized mean differences (SMD) with 95% confidence intervals (CI). In studies with more than one follow-up measurement, the follow-up measurement directly following the intervention was included in the meta-analysis. A random effects model was selected a priori to account for between-study variation in intervention protocol, duration, intensity, and participant characteristics. Statistical heterogeneity was evaluated using the *p*-value from the chi-square tests for heterogeneity and the I^2^ statistic [25]. The I^2^ statistic was interpreted as follows: 0–40% likely unimportant; 30–60% may represent moderate heterogeneity; 50–90% may represent substantial heterogeneity; and 75–100% considerable heterogeneity [25].

Random effects models were used in the meta-analyses to account for the fact that the various tests used different units of measurement (meters, seconds, reps, Watts). Furthermore, whereas an improvement measured in timed tests is shown by decreasing values (a 400-m walk test in 5 minutes is better than one in 7 minutes), an improvement in meters, repetitions, or Watt/kg is reflected in increasing values (10 repetitions of a chair rise is better than 8 repetitions). To account for this difference in measurement scales, the test outcomes of timed tests were switched between power training and strength training groups to adequately reflect the more superior intervention.

### Quality of evidence assessment

The PEDro scale (Physiotherapy Evidence Database, 1999) was used to assess the internal validity of each randomized controlled trial included in the systematic review. Trials with a score of 0–4 are considered ‘poor’, 4–5 ‘fair’, 6–8 ‘good’, and 9–10 ‘excellent’.

The quality of the evidence was assessed using the GRADEpro Guideline Development Tool (Evidence Prime, 2015). The initial GRADE score began as “high” because each of the selected studies were RCTs and was downgraded as a result of limitations with respect to risk of bias, inconsistency, indirectness, imprecision, or publication bias. The criteria for downgrading a level of evidence was based on the Grade Handbook [26] and the Cochrane Handbook [25]. Publication bias was additionally evaluated by visually assessing the distribution of effect sizes through funnel plot symmetry. In a symmetrical funnel plot the intercept on the X-axis should be close to 0, whereas with asymmetry it deviates considerably from 0 and suggests publication bias [27].

## Results

### Study selection

**Fig. 1:**
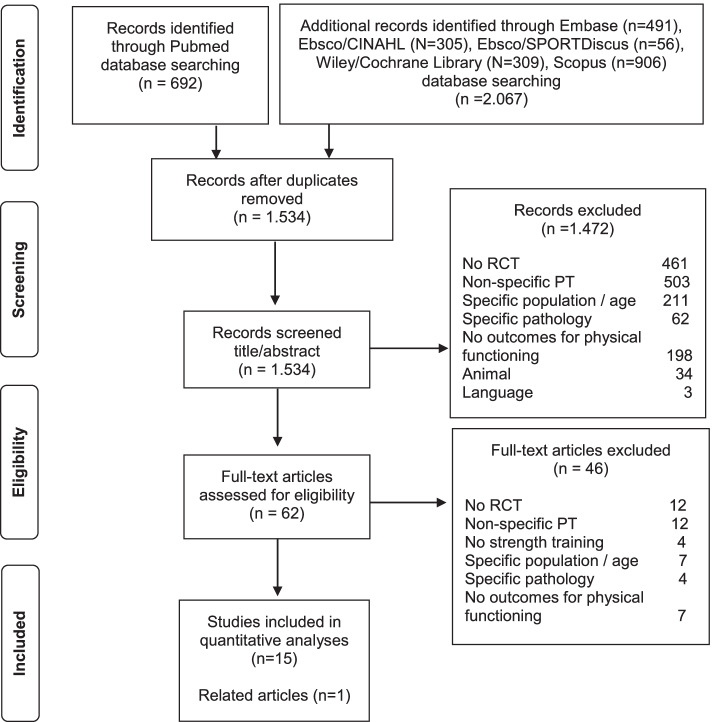
PRISMA flow diagram of literature search and study selection. Legend: RCT = randomized controlled trial; PT = power training; ST = strength training

The initial search yielded 2.759 articles, of which 1.534 entered preliminary screening after removal of duplicates. The screening of abstracts produced 62 full-text articles to be read in entirety for eligibility in phase 2. Forty-six studies were excluded and 16 studies were included. One of these studies [28] was excluded from quantitative analysis on the basis of using interquartile range (IQR) as a distribution parameter, which did not comply with the remaining 15 studies that were used in the meta-analyses. A PRISMA flow diagram of the literature search and study selection is shown in Fig. 1.

A total of 16 studies were included in the review. The total study population for the main comparison of power training versus strength training consisted of 583 participants, of which 277 participants received power training (47.5%). The study population for the secondary analysis of power training versus a non-training control group was 272, of which 134 participants received power training (49.3%).

### Outcome assessment

The selected studies used a variety of tests to measure muscle power and performance on activity tests. In the included studies, the construct of muscle power was captured with the chest press for upper extremity muscle power, and the leg press for lower extremity muscle power. Our analyses used the power and acceleration outcomes reported in the original studies. Tests with an emphasis on movement speed were tests in which the instruction was to perform the test “as quickly as possible”. The activity tests categorised as having an emphasis on movement speed were countermovement jump, chair rise (seconds), short physical performance battery, walking speed, timed up and go, timed stair climb, and timed floor rise to stand. Generic tests were tests for which there was no instruction regarding the rate of force development. The activity tests categorised as generic were the 6-minute walk test, 400-m walk test, chair rise, sit to stand transfer (Watt and repetitions), and a summary measure for balance. None of the included studies used the level of physical activity in daily life as an outcome.

The characteristics of the included studies included are shown in Table 1.

**Table 1:** Characteristics of the studies included in the meta-analyses evaluating muscle power, activity-based tests, and physical activity level in daily life in older adults

Study	Type	Frequency and duration	Intensity	N	Age, years (SD)	Sex, female	Relevant outcomes	Test used
Balachandran et al. (2014) [29]	PT	2x/wk. for 15 wks	50–80% 1RM	8	71.6 (7.8)	8 (100%)	Muscle power	Chest Press
	ST	2x/wk. for 15 wks	70% 1RM	9	71.0 (8.2)	8 (88%)		Leg Press
							Activity test: emphasis on movement speed	Chair rise (s)
								SPPB
								Stair climb (s)
Bean et al. (2009) [3]	PT	3x/wk. for 16 wks	11–16 RPE	59	74.7 (6.8)	50 (69%)	Muscle power	Leg press
	ST	3x/wk. for 16 wks	11–16 RPE	58	76.1 (6.9)	45 (68%)	Activity test: emphasis on movement speed	SPPB
Bottaro et al. (2007) [4]	PT	2x/wk. for 10 wks	40–60% 1RM	11	66.6 (5.8)	0 (0%)	Muscle power	Chest Press
	ST	2x/wk. for 10 wks	40–60% 1RM	9	66.3 (4.8)	0 (0%)		Leg Press
							Activity test: emphasis on movement speed	Timed up and go
							Activity test: generic tests	Chair raise (reps)
Cadore et al. (2013) [30]	PT	2x/wk. for 12 wks	40–60% 1RM	11	93.4 (3.2)	not reported	Activity test: emphasis on movement speed	Walking speed
	MT	4x/wk. for 12 wks	30 min/day	13	90.1 (1.1)	not reported		Timed up and go
							Activity test: generic tests	Chair raise (reps)
								Sit to stand transfer (W)
								Balance
Fielding et al. (2002) [31]	PT	3x/wk. for 16 wks	70% 1RM	15	73.2 (1.2)	15 (100%)	Muscle power	Leg press
	ST	3x/wk. for 16 wks	70% 1RM	15	72.1 (1.3)	15 (100%)		
Henwood et al. (2006) [5]	PT	2x/wk. for 8 wks	50–75% 1RM	21	70.7 (5.5)	14 (60%)	Muscle power	Chest press
	ST	2x/wk. for 8 wks	45–75% 1RM	20	70.2 (5.0)	11 (50%)	Activity test: emphasis on movement speed	Chair rise (s)
								Walking speed
								Stair climb (s)
								Floor rise to stand
							Activity test: generic tests	400 m walk test
Henwood et al. (2008) [32]	PT	2x/wk. for 24 wks	50–75% 1RM	19	71.2 (1.3)	12 (63%)	Muscle power	Chest press
	ST	2x/wk. for 24 wks	75% 1RM	19	69.6 (1.1)	12 (63%)		Leg press
							Activity test: emphasis on movement speed	Chair Rise (s)
								Walking speed
								Stair climb (s)
								Floor Rise to Stand (s)
							Activity test: generic tests	400 m Walk Test
Lopes et al. (2014) [33]	PT	3x/wk. for 12 wks	70–90% 1RM	11	63.3 (3.9)	not reported	Muscle power	Leg press
	ST	3x/wk. for 12 wks	70–90% 1RM	13	67.0 (6.1)	not reported		
Marsh et al. (2009) [34]	PT	3x/wk. for 12 wks	70% 1RM	12	76.8 (6.4)	7 (58%)	Muscle power	Leg press
	ST	3x/wk. for 12 wks	70% 1RM	11	74.6 (5.4)	9 (82%)	Activity test: emphasis on movement speed	SPPB
Miszko et al. (2003) [7]	PT	3x/wk. for 16 wks	50–80/40% 1RM	11	72.3 (6.7)	6 (55%)	Muscle power	Chest press
	ST	3x/wk. for 16 wks	50–80% 1RM	13	72.8 (5.4)	7 (54%)	Activity test: emphasis on movement speed	SPPB
							Activity test: generic tests	Balance
Orr et al. (2006) [35]	PT	2x/wk. for 10 wks	80% 1RM	24	69.0 (6.4)	17 (61%)	Muscle Power	Leg Press
	ST	2x/wk. for 10 wks	50% 1RM	25	68.1 (4.5)	17 (61%)	Activity test: generic tests	Balance
	ST	2x/wk. for 10 wks	20% 1RM	25	69.4 (5.8)	17 (61%)		
Ramirez-Campillo et al. (2014) [19]	PT	3x/wk. for 12 wks	45% - /75%	20	66.3 (3.7)	20 (100%)	Muscle Power	Chest press
	ST	3x/wk. for 12 wks	75% 1RM	20	68.7 (6.4)	20 (100%)		Leg press
							Activity test: emphasis on movement speed	CMJ (cm)
								Walking speed
								Timed up and go
							Activity test: generic tests	Chair raise (reps)
								Sit to stand transfer
Reid et al. (2013) [36]	PT	2x/wk. for 16 wks	40% 1RM	27	78.3 (5.0)	15 (56%)	Muscle power	Leg press
	ST	2x/wk. for 16 wks	70% 1RM	25	77.6 (4.0)	18 (72%)		
Tiggeman et al. (2016) [37]	PT	2x/wk. for 12 wks	45/55/65% 1RM	12	64.4 (4.0)	12 (100%)	Activity test: emphasis on movement speed	CMJ (cm)
	ST	2x/wk. for 12 wks	45/55/65% 1RM	13	65.6 (5.3)	13 (100%)		Chair rise (s)
								Timed up and go
								Stair climb (s)
							Activity test: generic tests	6 Min Walking test
Zech et al. (2012) [38]	PT	2x/wk. for 12 wks	10–16 RPE	16	77.4 (6.2)	not reported	Activity test: emphasis on movement speed	SPPB
	ST	2x/wk. for 12 wks	10–16 RPE	18	77.8 (6.1)	not reported	Activity test: generic tests	Sit to stand transfer
								Balance

*Legend: PT* power training, *ST* strength training,* MT* mobility training, *SPPB* Short Physical Performance Battery, *1RM* 1-repetition maximum, *RPE* rate of perceived exertion, *s* seconds, *reps* repetitions, *W* Watt

**Table 2:** GRADE quality of evidence table for estimates using muscle power, activity-based tests, and physical activity level in daily life in older adults

Certainty assessment							Number of patients		Effect		Certainty	Importance
Number of studies	Study design	Risk of bias	Inconsist-ency	Indirectness	Imprecision	Other considerations	Power training	Strength training	Relative (95% CI)	Absolute (95% CI)		
Muscle power (upper extremity)												
6	randomized trials	serious ^a^	not serious ^b,c,d^	not serious ^e,f,g^	not serious ^h^	all plausible residual confounding would suggest spurious effect, while no effect was observed	90	90	–	SMD **0.99 SD higher**(0.34 higher to 1.65 higher)	⨁⨁⨁⨁ HIGH	IMPORTANT
Muscle power (lower extremity)												
10	randomized trials	serious ^a^	very serious ^b,i,j^	not serious ^e,f,g^	not serious ^h^	publication bias strongly suspected very strong association all plausible residual confounding would suggest spurious effect, while no effect was observed	203	204	–	SMD **1.00 SD higher**(0.40 higher to 1.60 higher)	⨁⨁⨁◯ MODERATE	IMPORTANT
Generic tests												
11	randomized trials	serious ^a^	not serious ^b,c,j^	not serious ^e,f,g^	not serious ^h^	strong association all plausible residual confounding would suggest spurious effect, while no effect was observed	424	429	–	SMD **0.43 SD higher**(0.23 higher to 0.62 higher)	⨁⨁⨁⨁ HIGH	CRITICAL
Tests with emphasis on movement speed												
9	randomized trials	serious ^a^	not serious ^b,d,i^	not serious ^e,f,g^	not serious ^h^	strong association all plausible residual confounding would suggest spurious effect, while no effect was observed	202	215	–	SMD **0.36 SD higher**(0.04 higher to 0.6 higher)	⨁⨁⨁⨁ HIGH	IMPORTANT

Legend: ^a^ Studies that carried large weight for the overall effect estimated as high risk of bias due to lack of construct validity for the intervention and the test and a lack of allocation concealment ^b^ (Unexplained) Inconsistency, with point estimates different. ^c^ Substantially overlap in confidence interval ^d^ I^2^ is less than 60% ^e^ No differences in population, ^f^ Substantial differences in outcome measures ^g^ Substantial differences in interventions ^h^ The 95% CI showed a moderate to good effect of powertraining in all articles ^i^ Confidence intervals do not overlap. ^j^ I^2^ is more than 60%

### Risk of bias

**Fig. 2:**
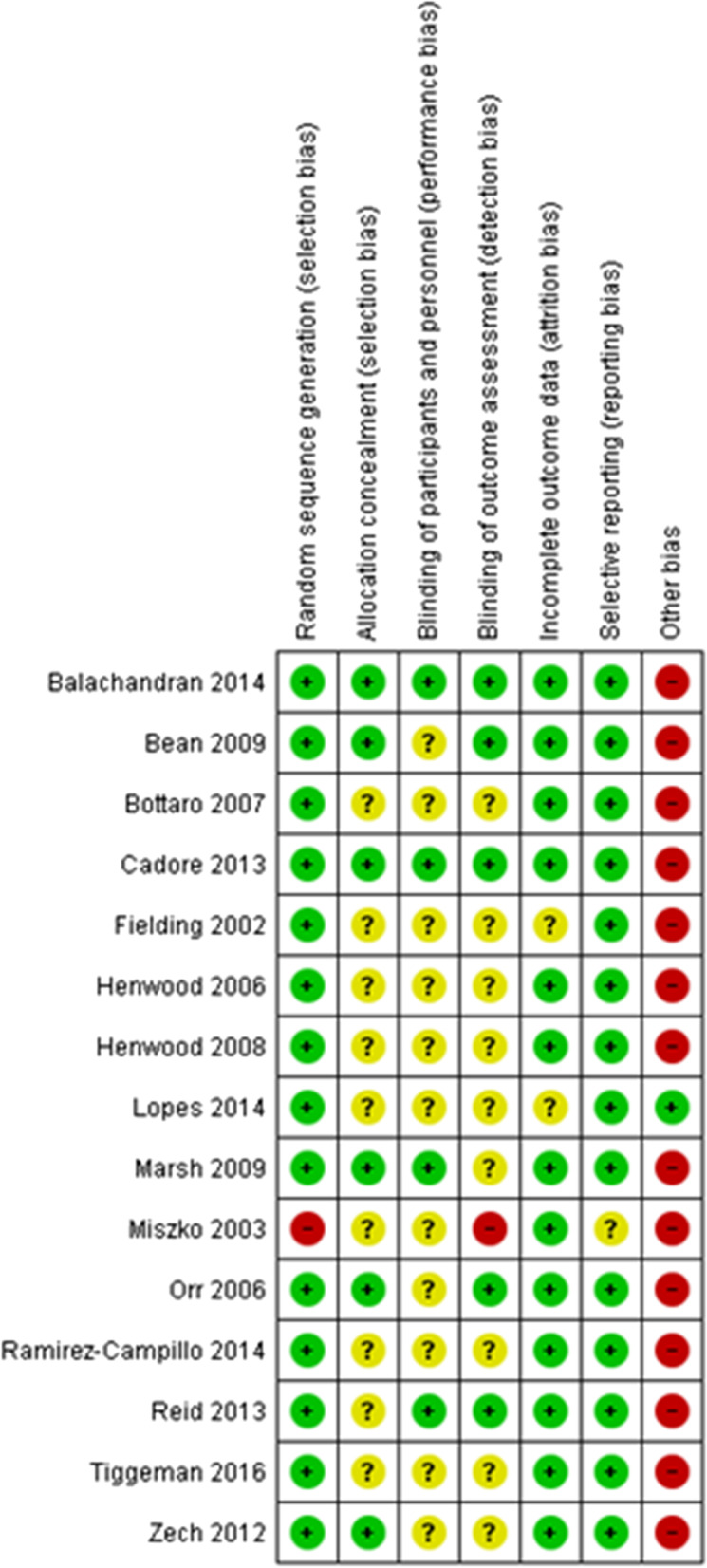
Risk of bias for the studies included in the meta-analysis

An overview of the risk of bias assessment for the included studies is provided in Fig. 2. In conclusion, 47% of the studies were considered to be low risk of bias [3, 29, 30, 34–36, 38] while the remaining studies were scored as being unclear to high [4, 5, 7, 19, 31–33, 37].

### Muscle power

**Fig. 3:**
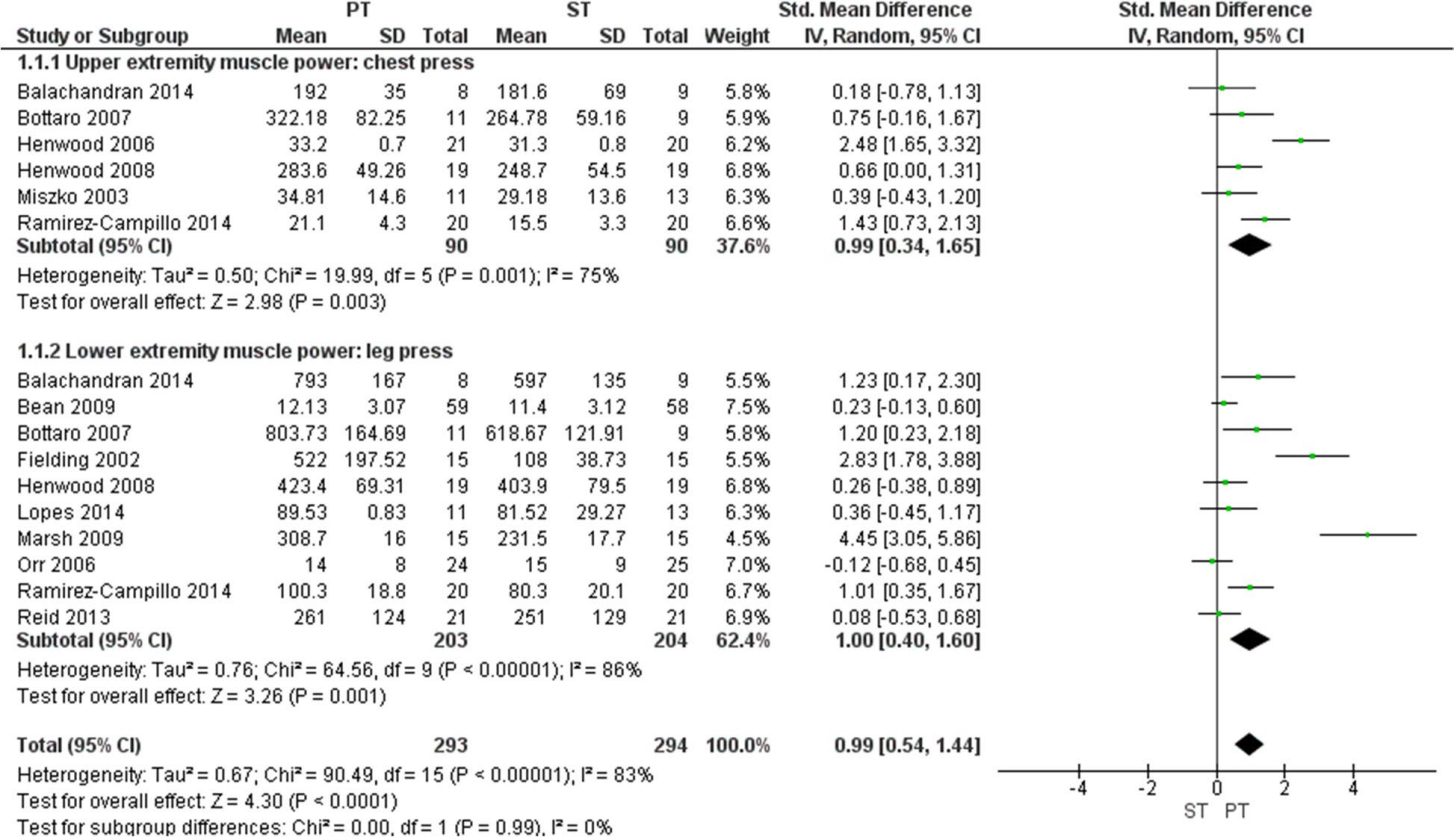
Forest plot comparing power training to strength training using muscle power. Legend: Forest plot showing standardized mean difference between power training and strength training in older adults according to chest press and leg press. PT = power training; ST = strength training; SD = standard deviation; IV = intravitreal; CI = confidence interval

The statistical analyses for muscle power as outcome was performed separately for the upper extremity (UE) and lower extremity (LE) (Fig. 3). For UE muscle power, a meta-analysis of 6 RCTs showed a significant benefit of power training compared to strength training (SMD: 0.99, 95% CI: 0.34 to1.65, *p* = 0.003). A significant chi-square test for heterogeneity (*p* = 0.001) indicates a concern for statistical heterogeneity, which was corroborated by an I^2^ statistic of 75% indicating substantial to considerable heterogeneity [25]. For LE muscle power, a meta-analysis of 10 RCTs showed a significant benefit of power training compared to strength training (SMD: 1.00, 95% CI: 0.40 to 1.60; *p* = 0.001). A significant chi-square test for heterogeneity (*p* < 0.001) indicates a concern for statistical heterogeneity, which was corroborated by an I^2^ statistic of 86% indicating considerable heterogeneity [25]. The overall combined effect for UE and LE muscle power clearly favors power training (SMD: 0.99, 95% CI: 0.54 to 1.44, *p* < 0.001) over strength training.

The secondary meta-analysis showed that power training was also significantly improved UE muscle power (SMD: 0.81, 95% CI: 0.35 to 1.27, *p* < 0.001) and LE muscle power (SMD: 1.38, 95% CI: 0.90 to 1.86, *p* < 0.001) compared to to not training (Additional file 2). Statistical heterogenity for the secondary analyses is likely ‘unimportant’ to ‘moderate’ [25]. The overall combined effect for UE and LE muscle power also favors power training (SMD: 1.12, 95% CI: 0.75 to 1.48, *p* < 0.001) over not training.

### Generic activity tests

**Fig. 4:**
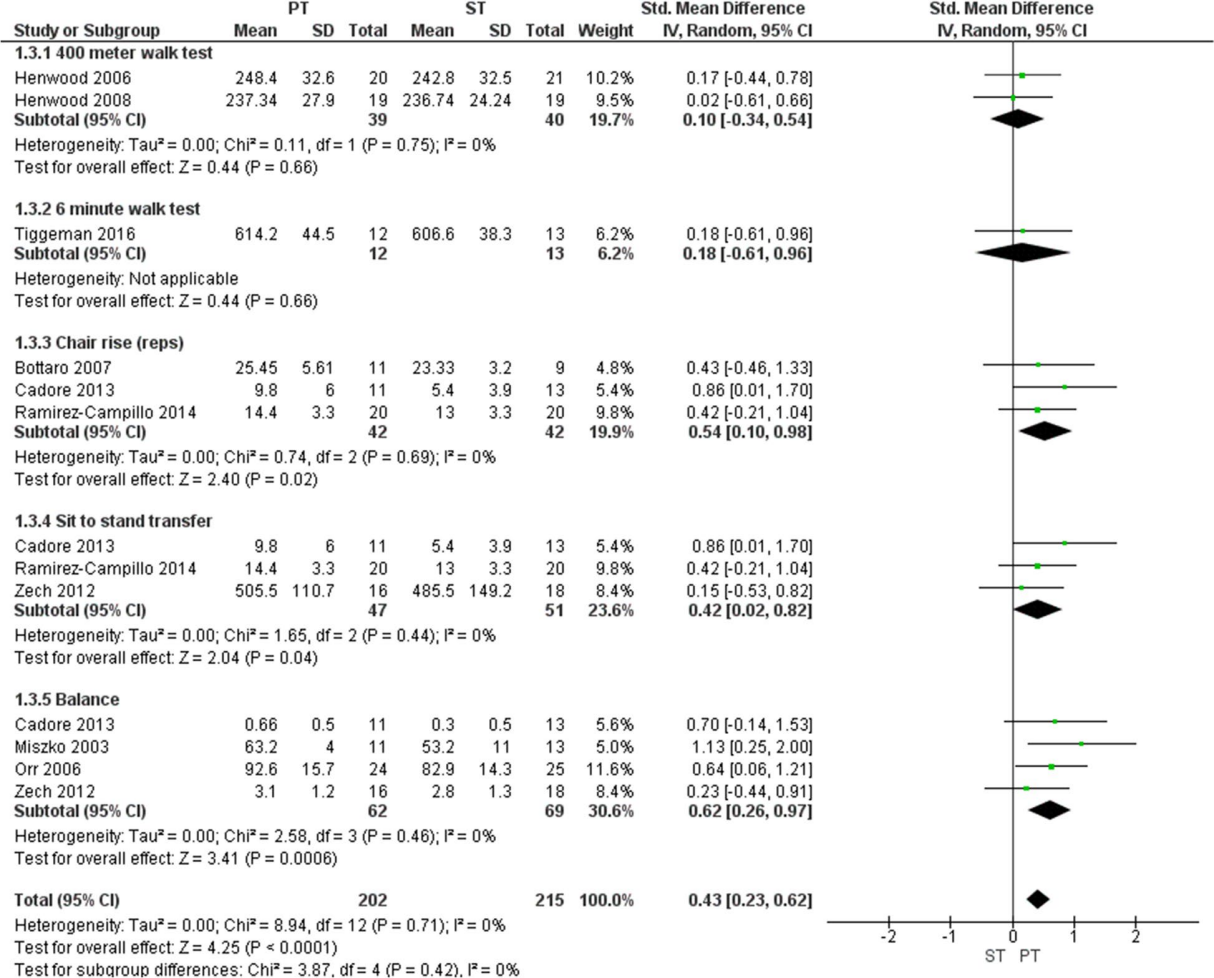
Forest plot comparing power training to strength training using generic tests. Legend: Forest plot showing standardized mean difference between power training and strength training in older adults according to 400 m walk test, 6 minute walk test, chair rise (reps), sit to stand transfer, and balance. The sit to stand transfer and the chair rise in Cadore et al. is, in fact, the same performance test but interpreted in two different manners. Sit to stand is considered to be a performance while chair rise is considered to be a physical function. PT = power training; ST = strength training; SD = standard deviation; IV = intravitreal; CI = confidence interval

Five different tests were used in this category. A subgroup analysis was performed for each test (Fig. 4). In the subgroup analyses, chair raise *(p =* 0.02), sit to stand transfer *(p =* 0.04), balance *(p <* 0.001), walking speed 400 m *(p =* 0.66), and 6-minute walk test *(p =* 0.66) all favored power training. The overall effect of functional performance was calculated by pooling the effects of all subgroup analyses, which showed a significant benefit of power training over strength training (SMD: 0.43, 95% CI 0.23 to 0.62, *p* < 0.001). Statistical heterogeneity was of no concern for the subgroup or overall analyses [25].

The secondary analysis showed that power training group also significantly improved the performance on the generic tests (SMD: 0.73, 95% CI 0.48 to 0.99; *p* < 0.001) compared to not training (Additional file 3). Within the subgroup analyses, chair raise (*p* < 0.001), sit to stand transfer (*p* = 0.02), balance (*p* = 0.003), and walking speed 400 m (*p* = 0.14) all favored power training. Statistical heterogeneity was of no concern in the overall analyses, however, the chi-square test for heterogeneity in walking speed 400 m (*p* = 0.01) and an I^2^ statistic of 84% indicated there may be substantial to considerate heterogeneity [25].

### Activity tests with an emphasis on movement speed

**Fig. 5:**
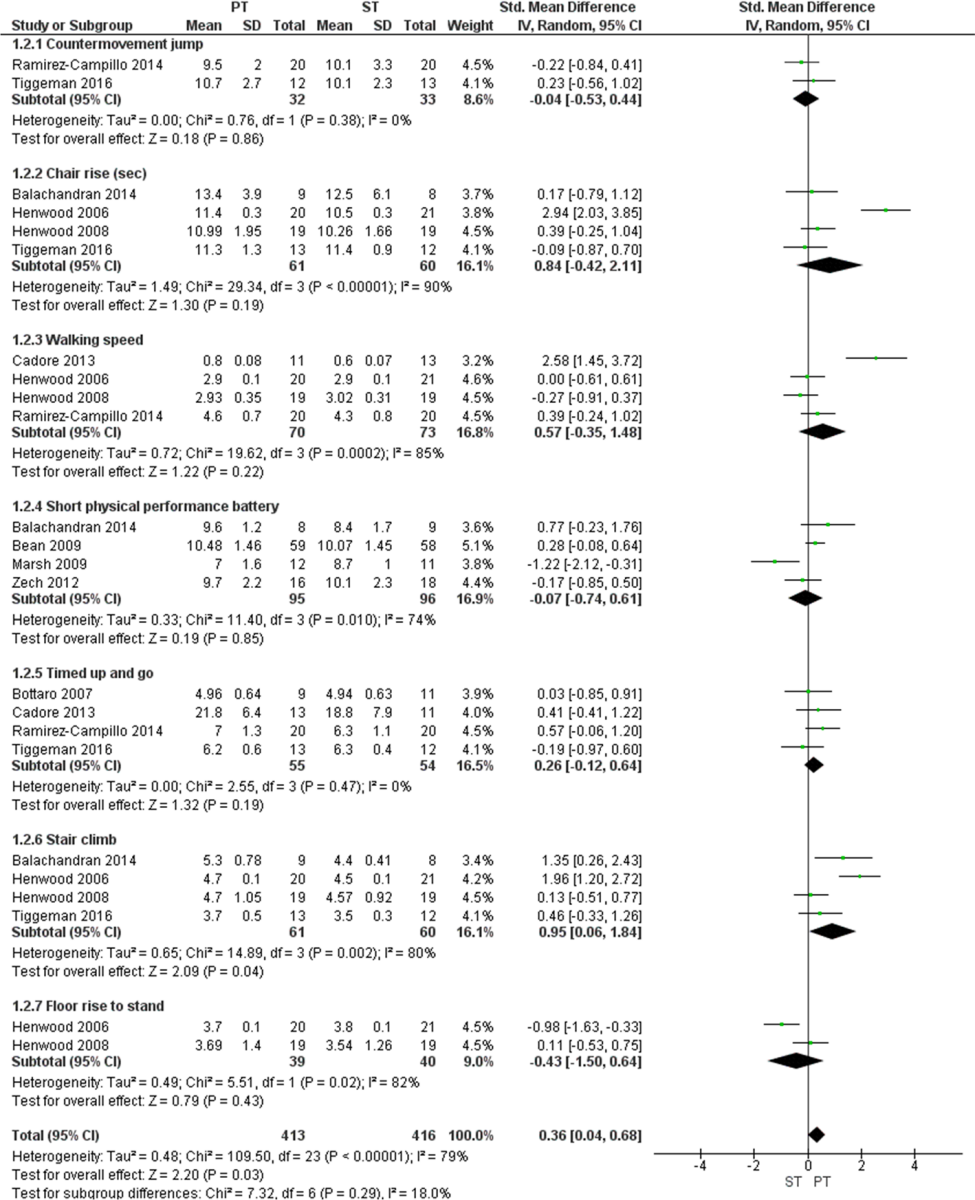
Forest plot comparing power training to strength training using tests with emphasis on movement speed. Legend: Forest plot showing standardized mean difference between power training and strength training in older adults according to the countermovement jump, chair rise, walking speed, short physical capacity battery, timed up and go, stair climb, and floor rise to stand. PT = power training; ST = strength training; SD = standard deviation; IV = intravitreal; CI = confidence interval

Seven different tests were used in this category, and a subgroup analysis was performed for each test (Fig. 5). Within the subgroup analyses, stair climb *(p =* 0.04), chair rise *(p =* 0.19), walking speed *(p =* 0.22), Short Physical Performance Battery (SPPB) *(p =* 0.75), and timed up and go *(p =* 0.19) favored power training, while the countermovement jump (CMJ) *(p =* 0.86) and floor rise to stand *(p =* 0.43) favored strength training. Statistical heterogeneity may be a cause for concern in chair rise, walking speed, SPPB, stair climb, and floor to rise stand, for which a significant *p*-value and an I^2^ statistic above 70% indicated substantial to considerable heterogeneity [25]. The overall effect was calculated by pooling the effects of each subgroup analysis, which showed a significant benefit of power training compared to strength training (SMD: 0.36, 95% CI 0.06 to 0.68, *p* = 0.02). Statistical heterogeneity may be cause for concern as well, as a significant chi-square test for heterogeneity *(p <* 0.001) and an I^2^ statistic of 78% indicate substantial to considerable heterogeneity [25].

The secondary analysis showed that power training also significantly improved performance on the speed tests (SMD: 0.74, 95% CI 0.50 to 0.98, *p* < 0.001) compared to not training (Additional file 4). A significant chi-square test for heterogeneity (*p* = 0.002) indicated a concern for statistical heterogeneity, which was corroborated by an I^2^ statistic of 46% indicating moderate heterogeneity [25]. Within the subgroup analyses, CMJ (*p* = 0.06), chair rise (*p* < 0.001), walking speed (*p* = 0.004), time up and go (*p* = 0.05), and stair climb (*p* = 0.03), SPPB (*p* = 0.32), and floor rise to stand (*p* = 0.07) all favored power training. The chi-square test for heterogeneity indicated a cause for concern in walking speed, for which the I^2^ statistic was 71% [25].

### Quality of evidence

The quality of evidence for each outcome is shown in the GRADE quality of evidence table (Table 2). The effect estimates for LE muscle power, generic and speed-based activity tests, were scored as having a ‘high’ amount of certainty, while UE muscle power was scored as having a ‘moderate amount’ of certainty. Within the certainty assessment, risk of bias was graded as ‘serious’ for each outcome, largely due to the lack of allocation concealment the selected studies. Indirectness was graded as ‘very serious’ for LE muscle power as a result of the inconsistent point estimates and high levels of statistical heterogeneity in the meta-analysis. For the activity tests with an emphasis on speed, criteria for indirectness (differences in outcome measures) and imprecision (risks of random errors) within the GRADE were scored as ‘not serious’.

Publication bias was also assessed separately for each outcome through the use of funnel plots (Additional file 5, Additional file 6 and Additional file 7). The funnel plot for muscle power is a-symmetrical, indicating that publication bias may be present. The funnel plots for the activity tests do not show asymmetry, indicating that publication bias is less of a concern. The PEDro score of each of the selected studies is illustrated in Additional file 8. Five studies were considered to have ‘good’ internal validity [3, 30, 33, 35, 38], 8 studies were classified as ‘poor’ to ‘fair’ [4, 5, 9, 29, 31, 32, 37, 39], and 1 study was classified as having ‘poor’ interal validity [7].

## Discussion

### Summary of main results

The results of this meta-analysis indicate a significant benefit of power training compared to strength training on muscle power and activity tests with SMD’s varying between 0.99 for muscle power, 0.43 for generic tests, and 0.36 for tests with an emphasis on movement speed. Compared to non-training control groups, the effect was even larger with SMD’s of 1.12 for muscle power, 0.73 for generic tests, and 0.74 for tests with an emphasis on movement speed. These results support the findings of previous intervention studies [3, 4, 7, 8–, 17–20] and systematic reviews [8, 20], both of which found power training to be more effective at improving physical and functional outcomes. No publications reporting the effect of muscle power training on physical activity level were found.

In a meta-analysis comparing power training with strength training, Tschopp et al. [8] reported an SMD of 0.42 for muscle power (95% CI − 0.02 to 0.85), whereas we found an SMD of 0.99 (95% CI: 0.54 to 1.44; *p* < 0.001). However, some of the studies included in the meta-analysis by Tschopp et al. were actually investigations of different intensities of power training rather than a comparison of power training against strength training. The heterogeneity between studies has consequences for their overall conclusion that power training interventions are more effective than strength training. The meta-analysis from Steib et al. [20] reported an SMD of 1.66 for muscle power (95% CI 0.08 to 3.24) compared to progressive resistance training. However, the authors deemed the level of evidence as ‘moderate’ due to the large between-study variation. Furthermore, Byrne et al. [40] found that muscle power was a superior predictor of functional performance compared to muscle strength, however, pooled effect estimates were not provided.

Although these reviews found a similar trend as the present study, their methodological limitations prevented a direct comparison between power training and strength training. The present study reviewed the literature on the effect of power training compared to strength training in older adults using clear differentiation between the constructs muscle power, activity based tests and physical activity level in daily life. Therefore, this review makes a unique contribution to the body of evidence for the effect of power training in older adults.

The present study is, to our knowledge, the most comprehensive systematic review on this topic to date. The literature search for the present study used broad inclusion criteria to obtain as many studies comparing power training with strength training as possible. Each of the selected studies was thoroughly and independently examined by two researchers. The method in which risk of bias and quality of evidence were determined in the selected studies used the highest quality guidelines for systematic reviews.

While the following was not part of eligibility criteria, it is relevant to note that all of the interventions from the selected studies were longer than 8 weeks in duration which complies with the the American College of Sports Medicine (ACSM) guidelines. In these guidelines a minimum duration of 6 weeks is recommended for a measurable improvement caused by increased neurological efficiency [41]. A period to 8-weeks is considered to be the minimum amount of time required for physiological changes in muscle structure and strength [42, 43].

The present study encountered several limitations that provide opportunities for future research. There was a substantial amount of variability in the activity based tests used to measure outcomes in each of the selected studies, making comparibility between some studies more difficult. As a result, the overall quality of evidence was downgraded for each comparison based on ‘indirectness’ and a random effects model was selected for the meta-analyses. Due to a lack of detailed information in the included studies, this review has not evaluated the protocols of the power and strength training interventions and, therefore, cannot guarantee that total workload, rest intervals, exercises prescribed, muscle groups stimulated and ranges of motion were comparable between power and strength training across the studies included. Future research should assess the validity of power and strength training interventions in older adults and future studies comparing power and strength training should provide more detailed information on training dosage.

Another limitation of the included studies is the lack of information about the degree of muscle power loss. Power training should ideally be targeted in persons with an established decline in muscle power. However, differences in baseline muscle power between participants of the included studies were not taken into account. Some studies included older adults in a geriatric care setting where muscle power is likely lower than in community-dwelling older adults. Other age- (young old versus oldest old), sex-, or ethnicity-related differences between participants were also not accounted for, but are important determinants of the decrease in muscle power with increasing age [11, 13, 44]. Because this review only included participants aged on average over 65 years, it is reasonable to assume that a decrease in muscle power as a result of aging had occurred, but it is also possible that in subgroups with a relative larger decline in muscle power the effect of power training is larger.

Another possibility for an underestimation of the effect of power training in our review, is the heterogeneity between the interventions themselves. Some of the interventions used in the selected studies trained muscle power using the low velocity and high force approach, as opposed to the high velocity and low force approach. Literature suggests that the in older adults, muscle power is best trained through the high velocity and low force approach. The degeneration of fast twitch fibers, which are responsible for explosive power, occurs more rapidly than the decrease of muscle strength [45]. Thus, age-related decrease in muscle power is determined by a decrease in velocity more than by a decrease in force produced, indicating that training should emphasize velocity more than force [10, 17]. The definition of power training used in the present study does not specify the load at which power training is to be performed, resulting in a combination of different power training intensities included in the meta-analysis. Literature suggests an ideal load of 20–30% of the 1- repetition maximum (1RM) when training muscle power in older adults [46–49]. The above could also be the reason for the smaller effects we found on speed based tests. A high velocity, low force approach can be expected to result in larger effects on speed based tests.

Despite our efforts to evaluate publication bias through the use of funnel plots, we acknowledge that asymmetry could also be caused by small sample sizes and poor methodological quality of the included studies [50]. Lastly, this systematic review compared post-intervention measurements directly following the intervention and did not include a follow-up period beyond what was required for post-intervention measurements. Therefore, no inferences can be made on the long-term effects of the interventions.

Several important themes emerged that could be beneficial for future research. Currently, there is no broadly accepted, validated and standardized and coherent test protocol for measuring muscle power, related activity tests, and physical activity in daily life. To ensure comparibility between studies, further research is required to develop such a testprotocol. Especially the lack of measurements of physical activity in everyday life is important. Neither an increase in muscle strength nor a better performance on activity tests guarantees an effect on a physical activity in daily life (participation domain). Future research evaluating the validity and reliability of power training parameters (frequency and intensity) is also important. This will provide further guidance on the best approach to power training (high or low velocity, low or high force). Additional factors that could influence the results of power training, such as age-, sex-, and ethnicity-related differences in muscle power and the role of drop-outs in exercise interventions in older adults, have te be taken into account in design and report of future studies.

## Conclusions

Power training offers more potential for improving muscle power and performance on activity based tests in older adults than strength training. The level of evidence for this comparison was rated as being moderate to high. Future research should focus on appropriate power training programs with correct training parameters and valid and reliable outcome measurements through the use of a standardised testing protocol.

## Supplementary Information

**Additional file 1.** PubMed search string used in literature search.

**Additional file 2.** Forest plot comparing power training to non-training control group using muscle power.Legend: Forest plot showing standardized mean difference between power training and non-training control group in older adults according to chest press and leg press. PT = power training; SD = standard deviation; IV = intravitreal; CI = confidence interval.

**Additional file 3.** Forrest plot comparing power training to non-training control group using generic tests. Legend: Forest plot showing standardized mean difference between power training and non-training control group in older adults according to the 400 m walk test, 6 minute walk test, chair rise (reps), sit to stand transfer, and balance. The sit to stand transfer and the chair rise in Cadore et al. is, in fact, the same performance test but interpreted in two different manners. Sit to stand is considered to be a performance while chair rise is considered to be a physical function. PT = power training; SD = standard deviation; IV = intravitreal; CI = confidence interval.

**Additional file 4.** Forest plot comparing power training to non-training control group using tests with emphasis on movement speed. Legend: Forest plot showing standardized mean difference between power training and non-training control group in older adults according to the countermovement jump, chair rise, walking speed, short physical performance battery, timed up and go, stair climb, and floor rise to stand. PT = power training; SD = standard deviation; IV = intravitreal; CI = confidence interval.

**Additional file 5.** Funnel plot comparing power training to strength training in older adults using muscle power.

**Additional file 6.** Funnel plot comparing power training to strength training in older adults using generic tests.

**Additional file 7.** Funnel plot comparing power training to strength training in older adults using tests with an emphasis on movement speed.

**Additional file 8.** PEDro score for the studies included in the meta-analyses.

## Data Availability

Data sharing is not applicable to this article as no datasets were generated or analysed during the current study.

## Abbreviations

PRISMA: Preferred Reporting Items for Systematic Reviews and Meta-Analyses
RCT: randomized controlled trial
WHO: World Health Organization
SMD: standardized mean difference
CI: confidence interval
UE: upper extremity
LE: lower extremity
SPPB: Short Physical Performance Battery
CMJ: countermovement jump
ACSM: American College of Sports Medicine
1RM: 1-repetition maximum
HRC: Health Research Consultancy
