# Supplementary material for: Effectiveness of power training compared to strength training in older adults: a systematic review and meta-analysis
Source: Eur Rev Aging Phys Act. 2022 Aug 11;19:18. doi: 10.1186/s11556-022-00297-x (PMC9367108; doi:10.1186/s11556-022-00297-x)
Supplement: Supplementary file 1 — Additional file 1. PubMed search string used in literature search. [file 11556_2022_297_MOESM1_ESM.docx]

**Additional file 1.** PubMed search string used in literature search

(("Aged"[Mesh] OR "Aging"[Mesh] OR "Homes for the Aged"[Mesh] OR "Housing for the Elderly"[Mesh] OR "Geriatrics"[Mesh] OR "Geriatric Nursing"[Mesh] OR "Health Services for the Aged"[Mesh] OR (elder*[tw] OR eldest[tw] OR frail*[tw] OR geriatri*[tw] OR old age*[tw] OR oldest old*[tw] OR senior*[tw] OR senium[tw] OR very old*[tw] OR septuagenarian*[tw] OR octagenarian*[tw] OR octogenarian*[tw] OR nonagenarian*[tw] OR centarian*[tw] OR centenarian*[tw] OR supercentenarian*[tw] OR older people[tw] OR older subject*[tw] OR older patient*[tw] OR older age*[tw] OR older adult*[tw] OR older man[tw] OR older men[tw] OR older male*[tw] OR older woman[tw] OR older women[tw] OR older female*[tw] OR older population*[tw] OR older person*[tw]) OR aging[tiab] OR ageing[tiab] OR community-dwell*[tiab]) AND ("Self Efficacy"[Mesh] OR "Activities of Daily Living"[Mesh] OR "Quality of Life"[Mesh] OR self efficac*[tiab] OR "activities of daily living"[tiab] OR "activity of daily living"[tiab] OR "activities of daily life"[tiab] OR "activity of daily life"[tiab] OR daily living activit*[tiab] OR daily life activit*[tiab] OR adl[tiab] OR iadl[tiab] OR "quality of life"[tiab] OR life qualit*[tiab] OR living qualit*[tiab] OR "quality of living"[tiab] OR "qol"[tiab] OR "hrql"[tiab] OR "hrqol"[tiab] OR functional abilit*[tiab] OR physical abilit*[tiab] OR functional perform*[tiab] OR physical perform*[tiab] OR functional independen*[tiab] OR physical independen*[tiab] OR "Personal Autonomy"[Mesh] OR functional autonom*[tiab] OR physical autonom*[tiab] OR "Physical Fitness"[Mesh] OR Independent living[tiab] OR Living independent*[tiab] OR high function*[tiab] OR higher function*[tiab] OR physical function*[tiab]) AND ((power[tiab] OR plyometric*[tiab] OR high velocit*[tiab] OR high intens*[tiab] OR explosive strength[tiab]) AND ("Exercise"[Mesh] OR "Sports"[Mesh] OR "Exercise Therapy"[Mesh] OR training[tiab] OR physical activity[tiab] OR exercise[tiab]))) AND (randomized controlled trial[pt] OR controlled clinical trial[pt] OR randomized[tiab] OR randomised[tiab] OR placebo[tiab] OR randomly[tiab] OR trial[tiab] OR groups[tiab])
