# Supplementary material for: Effectiveness of power training compared to strength training in older adults: a systematic review and meta-analysis
Source: Eur Rev Aging Phys Act. 2022 Aug 11;19:18. doi: 10.1186/s11556-022-00297-x (PMC9367108; doi:10.1186/s11556-022-00297-x)
Supplement: Supplementary file 2 — Additional file 2. Forest plot comparing power training to non-training control group using muscle power.Legend: Forest plot showing standardized mean difference between power training and non-training control group in older adults according to chest press and leg press. PT = power training; SD = standard deviation; IV = intravitreal; CI = confidence interval. [file 11556_2022_297_MOESM2_ESM.docx]

| 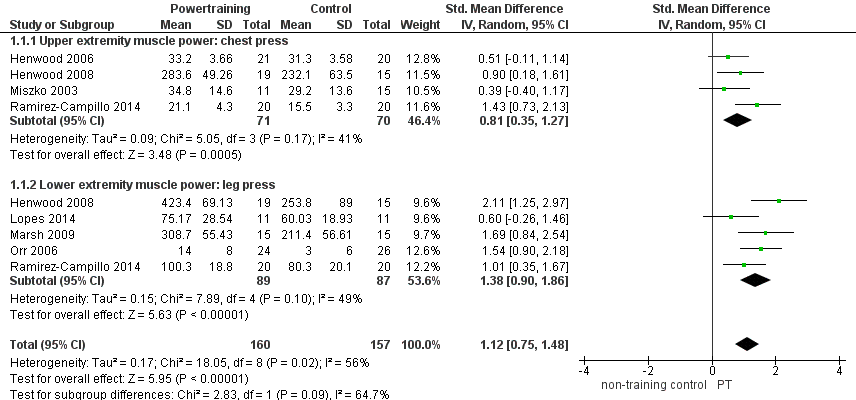 |
| --- |

**Additional file 2.** Forest plot comparing power training to non-training control group using muscle power.

Legend: Forest plot showing standardized mean difference between power training and non-training control group in older adults according to chest press and leg press. PT=power training; SD=standard deviation; IV=intravitreal; CI=confidence interval.
