# Supplementary material for: Effectiveness of power training compared to strength training in older adults: a systematic review and meta-analysis
Source: Eur Rev Aging Phys Act. 2022 Aug 11;19:18. doi: 10.1186/s11556-022-00297-x (PMC9367108; doi:10.1186/s11556-022-00297-x)
Supplement: Supplementary file 3 — Additional file 3. Forrest plot comparing power training to non-training control group using generic tests. Legend: Forest plot showing standardized mean difference between power training and non-training control group in older adults according to the 400 m walk test, 6 minute walk test, chair rise (reps), sit to stand transfer, and balance. The sit to stand transfer and the chair rise in Cadore et al. is, in fact, the same performance test but interpreted in two different manners. Sit to stand is considered to be a performance while chair rise is considered to be a physical function. PT = power training; SD = standard deviation; IV = intravitreal; CI = confidence interval. [file 11556_2022_297_MOESM3_ESM.docx]

*
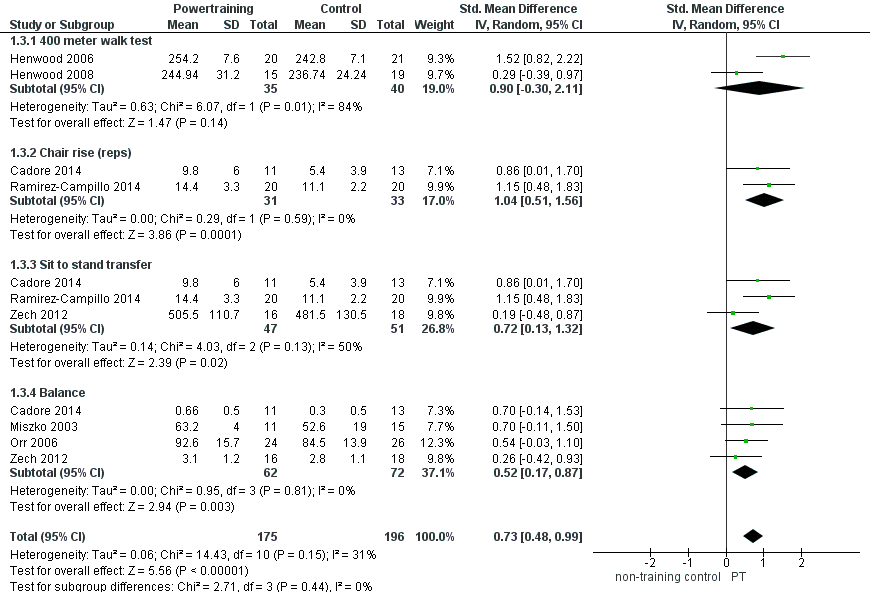
*

**Additional file 3.** Forrest plot comparing power training to non-training control group using generic tests.

Legend: Forest plot showing standardized mean difference between power training and non-training control group in older adults according to the 400 meter walk test, 6 minute walk test, chair rise (reps), sit to stand transfer, and balance. The sit to stand transfer and the chair rise in Cadore et al. is, in fact, the same performance test but interpreted in two different manners. Sit to stand is considered to be a performance while chair rise is considered to be a physical function. PT=power training; SD=standard deviation; IV=intravitreal; CI=confidence interval.
