# Supplementary material for: Effectiveness of power training compared to strength training in older adults: a systematic review and meta-analysis
Source: Eur Rev Aging Phys Act. 2022 Aug 11;19:18. doi: 10.1186/s11556-022-00297-x (PMC9367108; doi:10.1186/s11556-022-00297-x)
Supplement: Supplementary file 4 — Additional file 4. Forest plot comparing power training to non-training control group using tests with emphasis on movement speed. Legend: Forest plot showing standardized mean difference between power training and non-training control group in older adults according to the countermovement jump, chair rise, walking speed, short physical performance battery, timed up and go, stair climb, and floor rise to stand. PT = power training; SD = standard deviation; IV = intravitreal; CI = confidence interval. [file 11556_2022_297_MOESM4_ESM.docx]

| 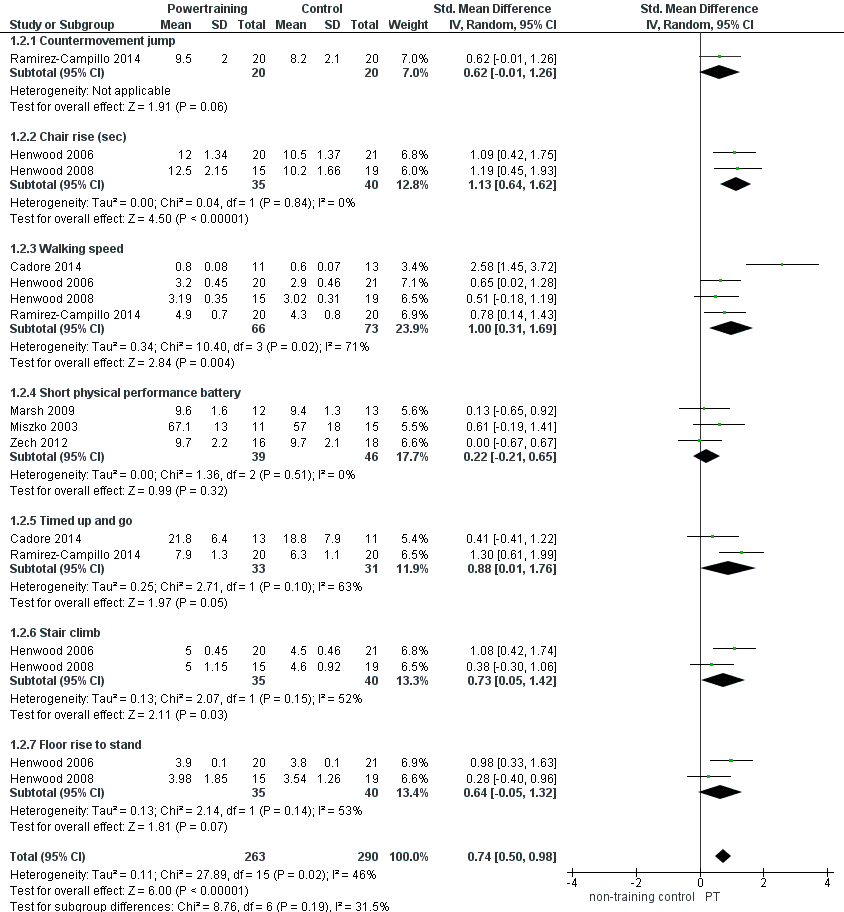 |
| --- |

**Additional file 4.** Forest plot comparing power training to non-training control group using tests with emphasis on movement speed.

Legend: Forest plot showing standardized mean difference between power training and non-training control group in older adults according to the countermovement jump, chair rise, walking speed, short physical performance battery, timed up and go, stair climb, and floor rise to stand. PT=power training; SD=standard deviation; IV=intravitreal; CI=confidence interval.
