# Supplementary material for: Effectiveness of power training compared to strength training in older adults: a systematic review and meta-analysis
Source: Eur Rev Aging Phys Act. 2022 Aug 11;19:18. doi: 10.1186/s11556-022-00297-x (PMC9367108; doi:10.1186/s11556-022-00297-x)
Supplement: Supplementary file 5 — Additional file 5. Funnel plot comparing power training to strength training in older adults using muscle power. [file 11556_2022_297_MOESM5_ESM.docx]

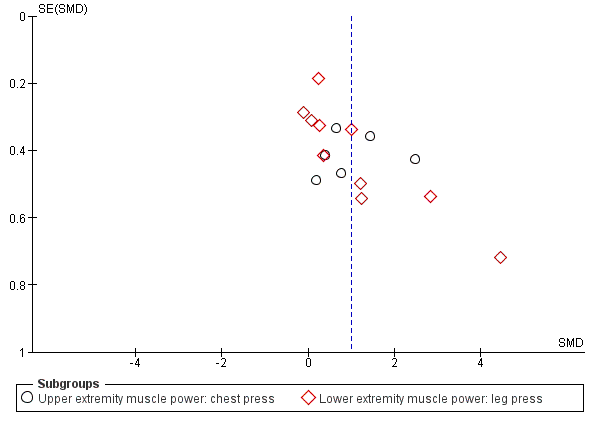


**Additional file 5.** Funnel plot comparing power training to strength training in older adults using muscle power.
