# Supplementary material for: Effectiveness of power training compared to strength training in older adults: a systematic review and meta-analysis
Source: Eur Rev Aging Phys Act. 2022 Aug 11;19:18. doi: 10.1186/s11556-022-00297-x (PMC9367108; doi:10.1186/s11556-022-00297-x)
Supplement: Supplementary file 7 — Additional file 7. Funnel plot comparing power training to strength training in older adults using tests with an emphasis on movement speed. [file 11556_2022_297_MOESM7_ESM.docx]

**
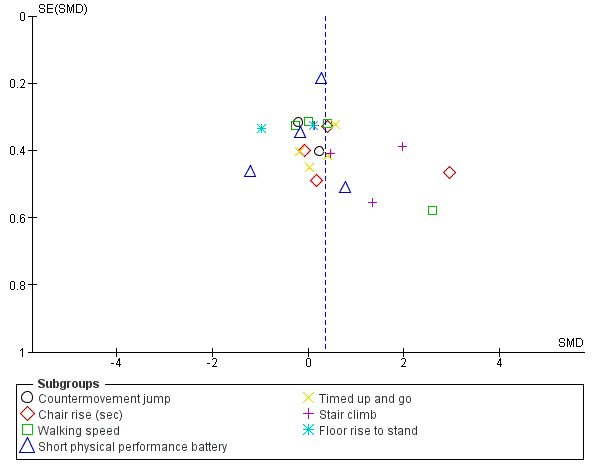
**

**Additional file 7.** Funnel plot comparing power training to strength training in older adults using tests with an emphasis on movement speed.
