# Supplementary material for: Effectiveness of power training compared to strength training in older adults: a systematic review and meta-analysis
Source: Eur Rev Aging Phys Act. 2022 Aug 11;19:18. doi: 10.1186/s11556-022-00297-x (PMC9367108; doi:10.1186/s11556-022-00297-x)
Supplement: Supplementary file 8 — Additional file 8. PEDro score for the studies included in the meta-analyses. [file 11556_2022_297_MOESM8_ESM.docx]

**Additional file 8.** PEDro score for the studies included in the meta-analyses.

| **Study** | **PEDro score (out of 10)** |
| --- | --- |
| Balachandran et al. (2014) | 5 |
| Bean et al. (2009) | 6 |
| Bottaro et al. (2007) | 4 |
| Cadore et al. (2013) | 6 |
| Fielding et al. (2002) | 4 |
| Henwood et al. (2006) | 5 |
| Henwood et al. (2008) | 4 |
| Lopes et al. (2014) | 6 |
| Marsh et al. (2009) | 4 |
| Miszko et al. (2003) | 3 |
| Orr et al. (2006) | 6 |
| Ramirez-Campillo et al. (2014) | 4 |
| Reid et al. (2013) | 6 |
| Tiggeman et al. (2016) | 4 |
| Zech et al. (2012) | 7 |
